# Opportunities for caries prevention using an ion-releasing coating material: a randomised clinical study

**DOI:** 10.1007/s10266-020-00551-7

**Published:** 2020-09-04

**Authors:** Ulf Örtengren, Anna Lehrkinder, Aram Safarloo, Jasmine Axelsson, Peter Lingström

**Affiliations:** 1 Department of Cariology, Institute of Odontology, Sahlgrenska Academy, University of Gothenburg, Box 450, 405 30 Göteborg, Sweden; 2 Department for Clinical Odontology, Faculty of Health Sciences, The Arctic University of Norway, Tromsø, Norway

**Keywords:** Caries prevention, Cariogenic bacteria, Coating, Dental materials, Ions

## Abstract

Ion-releasing materials (containing fluoride and boron, for example) have shown caries-preventive effects in vitro. The purpose of the present study was to investigate the impact of multi-ion-releasing coating material on pH stabilisation, plaque accumulation and the bacterial composition of dental plaque during a time period of 90 days. The null hypothesis tested here was that the evaluated material would not show any differences in pH stabilisation, plaque accumulation or bacterial composition compared with control material.

The study was carried out as a double-blind, split-mouth, randomised, controlled clinical trial in 28 volunteers. Over the evaluation period (days 4, 30, 60 and 90), pH measurements, plaque index and plaque sampling for bacterial analyses were conducted in a calibrated, standardized manner. The study received ethical permission and was carried out in accordance with the Helsinki Declaration.

A significant difference was observed, with less plaque accumulation over time in the subjects in whom the ion-releasing material was applied in comparison to the non-active group. No significant difference was evident in terms of either pH stabilisation or plaque levels of *mutans streptococci*.

The null hypothesis relating to plaque accumulation was rejected, with a lower plaque index shown for the test group up to 60–90 days. No adverse effects during the observation period were observed. Since the studied cohort was healthy from a caries perspective, more clinical studies are needed to further evaluate the caries-prevention potential of the ion-releasing material in other patient groups.

## Introduction

As our understanding of the caries disease and awareness of its preventive strategies are both increasing, the potential for a larger number of teeth to be retained at an older age has increased [1]. In spite of this, with advanced age, the potential for gingival retractions will be seen to a greater extent, thereby increasing the risk of a larger number of exposed root surfaces vulnerable to caries development.[2]. Dental caries is a demineralisation process and the result of an interplay between the tooth, cariogenic microflora and fermentable carbohydrates [3]. The pH response of the dental biofilm after a sugar challenge can be considered to mirror the acidogenic potential and thereby the risk of caries occurring [3–5]. The demineralisation process is known to start when pH is reduced below the critical pH (enamel ≈ 5.5–5.7, dentine ≈ 6.2) [3]. The onset of caries may be prevented if the pH during and after food intake is kept above the critical level. Important general precautionary strategies to prevent a destructive pH decrease and strengthen the enamel and dentine include changes in habits relating to sugar restriction, fluoride treatment and attempts to optimise oral hygiene [6]. In spite of this, an increase in the number of retained teeth with exposed root surfaces in the elderly will result in a larger number of unprotected areas subjected to demineralisation [2]. Furthermore, dental treatment, such as treatment with fixed orthodontic appliances, can create retention sites for cariogenic bacteria, with a risk of the increased local demineralisation of enamel. It has, therefore, been suggested that protecting the enamel and exposed dentine using additional strategies, such as applying materials with acid-neutralising features and the capacity to remineralise, is beneficial [7].

Fluoride is the most commonly used ion, as it promotes a more acid-resistant tooth surface due to its capability to convert the remineralisation of hydroxyapatite to fluorapatite by replacing Ca^2+^ with F^−^ [8]. Glass ionomers releasing NaF, due to the chemical reaction between carboxyl acids and active filler particles in the presence of water, have shown remineralisation properties. They have also been suggested to have antimicrobial properties as a result of the fluoride release [8]. Due to the development of materials, such as bioactive glasses with other ion-releasing properties than F^−^, additional caries-inhibiting properties have been suggested [9, 10]. As a result, ions such as strontium (Sr^2+^) and boron (B^3+^), with remineralising, antimicrobial and increased acid-resistant properties, have been identified [10]. Sr has shown positive effects in vitro on enamel and dentine due to its ability to convert hydroxyapatite into strontium apatite, with increased acid resistance as a result [11–13]. B has not only shown antimicrobial properties when added to antimicrobial agents for periodontal treatment, but it has also been suggested to be effective in caries prevention [14, 15].

**Fig. 1:**
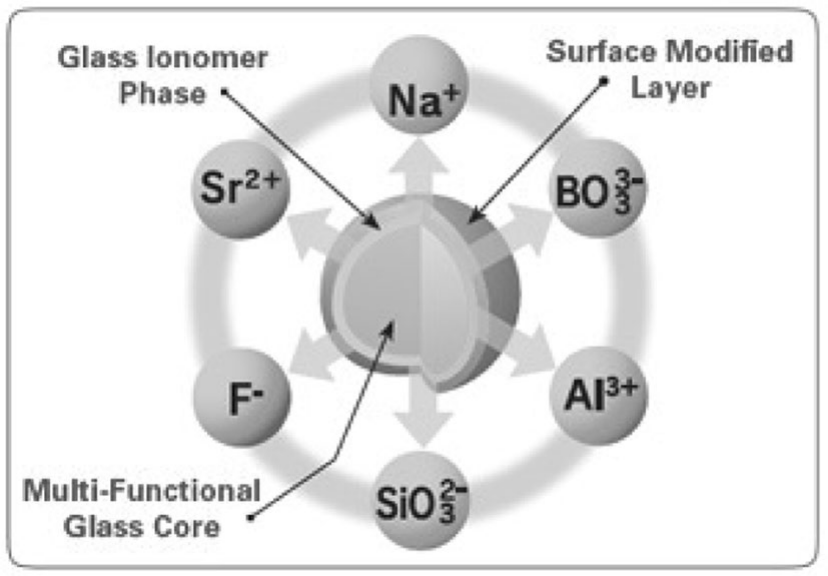
Structure of S-PRG particle (with permission from Shofu Inc.)

The concept of incorporating Surface Pre-Reacted Glass-ionomer (S-PRG) fillers with multi- ion-releasing properties in filling materials has been a focal area for the last few years. Due to the chemistry of the S-PRG filler, there have been attempts to combine the advantages of the active glass-ionomer fillers with those of bioactive glasses [10, 11]. The particles are composed of a three-layer structure with a suggested ability to release six ions (i.e., Na^+^, BO_3_^3−^, Al^3+^, SiO_3_^2−^, F^−^ and Sr^2+^) while maintaining the properties of the material (Fig. 1).

The S-PRG have been incorporated in composite resin-based materials but recently also in a light-curing ion-releasing coating material (PRG Barrier Coat, Shofu Inc., Fukuine, Higashiyama-Ku, Kyoto, Japan). The indications for the material, as given by the manufacturer, are presented in Table 1. The ion release has been confirmed in several studies and the material has been shown in vitro to neutralise acids, prevent the demineralisation of enamel, decrease plaque accumulation and change the bacterial composition of dental biofilm [8–11, 16]. *In-vitro* studies of the ion-releasing coating material, S-PRG Barrier Coat, have revealed positive arresting effects on factors important for caries development [10].

**Table 1:** Indications for S-PRG Barrier Coat

*Indications*	*Specific target areas*
Areas difficult to brush	Areas surrounding orthodontic brackets Difficult-to-brush areas due to crowded teeth Areas surrounding clasps
High caries risk areas	Exposed root surfaces Newly erupted molars White spot lesions
Areas of hypersensitivity	–

### Aim

To increase our knowledge of the caries-prevention effects of the material in vivo*,* a randomised, controlled clinical trial was designed*.* The aim was to investigate the impact of the ion-releasing coating material (S-PRG barrier coat) in comparison to a non-active product on pH stabilisation, plaque accumulation and bacterial composition in dental plaque over a period of 90 days. The formulated null hypothesis was that the active coating material applied to tooth surfaces would not reveal any differences in pH stabilisation over time or plaque accumulation or the bacterial composition of the dental biofilm in comparison to controls.

## Materials and methods

The study was designed as a double-blind, randomised, placebo-controlled (the placebo is hereinafter referred to as the non-active group), split-mouth study of 28 subjects. It was performed at the Department of Cariology, Institute of Odontology, Sahlgrenska Academy, University of Gothenburg, Sweden. The study was reviewed and approved by the regional ethical review board in Gothenburg, Sweden (1174-1116).

### Enrolment

The patients were enrolled using the following criteria*.* The inclusion criteria were: (1) age > 18 years, (2) normal stimulated salivary secretion rate (≥ 0.7 ml/min), (3) ≥ 2 teeth without restorations on the buccal surface and (4) signed informed consent. The following exclusion criteria were used: (1) age < 18 years, (2) reduced stimulated salivary secretion rate (< 0.7 ml/min), (3) allergy to composite materials, (4) pregnancy, (5) antibiotic use during the last 4 weeks prior to the study period and (6) subjects undergoing other dental treatment throughout the study period. All the subjects who met the inclusion criteria obtained written and detailed information about the study and signed a written consent form. Reasons which may result in the premature withdrawal of an individual test subject included: (1) retraction of consent, (2) continued participation in the study no longer acceptable or justified on important medical grounds, (3) occurrence of intolerable adverse events, (4) violation of the protocol, (5), non-attendance by the test subject and (6) lack of compliance and motivation on the part of the test subject. The criteria for discontinuing the entire study were the recognition of new information and/or risks which require the benefit/risk ratio to be reassessed.

### Allocation and intervention

**Fig. 2:**
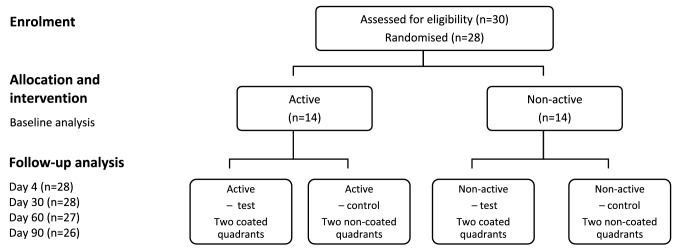
Flow chart of the design of the study

Twenty-eight volunteers (4 men and 24 women) met the criteria and were included in the study. They were randomly assigned to an active and a non-active group with 14 subjects in each group, using Microsoft^®^ Excel (v15.32, Microsoft Corp, Redmond, US), and each individual was coded (Fig. 2). The mean age of the active and non-active groups was 32.6 ± 9.2 and 32.2 ± 12.8 years, respectively.

The subjects made six visits to the research clinic as follows: a screening visit, a baseline visit (day 0) and follow-up visits on days 4, 30, 60 and 90. At the screening visit, the patients’ medical history was recorded and a routine dental examination, together with an assessment of unstimulated/stimulated salivary secretion rate and buffer capacity, was performed. Sites with no buccal fillings were selected for the application of PRG Barrier Coat or the non-active material (Shofu Inc., Fukuine, Higashiyama-Ku, Kyoto, Japan). The baseline visit (day 0) included: (i) an evaluation of laboratory and clinical parameters (plaque-pH registration, plaque index registration, stimulated saliva collection for levels of mutans streptococci and lactobacilli, plaque sampling for bacterial culturing and qPCR analysis) and (ii) the application of the barrier coat or non-active material, depending on the group assignment, as described below. The follow-up visits on days 4, 30, 60 and 90 after the intervention included: (i) an assessment of clinical symptomatology and (ii) an evaluation of laboratory and clinical parameters (as described above).

### Application of coating material

In the active and non-active group, the active or non-active coating materials, respectively, were applied to tooth surfaces in the 1st and 3rd quadrants or the 2nd and 4th quadrants; randomly assigned (Microsoft^®^ Excel, v15.32 Microsoft Co. Redmond, US). Two quadrants thus served as active/non-active test sites and the other two as respective controls. The coating materials were applied to two surfaces in each selected quadrant (molars and/or premolars).

The two materials that were applied were identical apart from the filler particles that were used. The active material contained S-PRG fillers, while the non-active material contained only pure silica (SiO_2_). The materials were stored at 4 ± 1 °C until used and were applied according to the manufacturer’s instructions as follows: (1) the tooth surface was cleaned with a rubber cup and polishing paste (Pressage, Shofu Inc., Fukuine, Higashiyama-Ku, Kyoto, Japan), after which the surface was thoroughly dried with air, (2) the base and active parts were mixed in the container using Dispotip (Shofu, Inc., Fukuine, Higashiyama-Ku, Kyoto, Japan), (3) a thin layer of the mixture was applied to the dried tooth surface and left undisturbed for more than 3 s (a maximum of three teeth were coated with a single container of the mixture), (4) each tooth was light cured for 10 s (performed within 2 min after the completion of mixing) and (5) after light-curing, any uncured layer was removed by gently rubbing the surface with a water-moistened cotton ball.

The participants were instructed not to brush their teeth for 48 h before each test session. They were not allowed to eat, drink or use any tobacco or chewing gum 1 h before baseline and each test session, as well as the hour following each application. Standard toothpaste containing 1450 ppm fluoride (Folktandkräm, Proxident AB, Falun, Sweden) was used by all volunteers throughout the study. The subjects used their regular toothbrushes and performed the normal oral hygiene procedures. No other fluoride products were allowed throughout the study.

### Calibration of investigators

The participating investigators were trained by the principal investigator regarding plaque acidogenicity measurements and the scoring of the amount of plaque prior to the study in an attempt to ensure uniform scoring.

### Plaque amount

The amount of plaque on each tooth (a total of eight teeth) was measured on three buccal sites (mesio-buccal, buccal, disto-buccal) and the plaque score was calculated using the index described by Silness and Löe [17]. Each tooth was scored from 0–3.

### Plaque acidogenicity evaluation

Dental plaque acidogenicity was measured at baseline (0 min) and 2, 5, 10, 15, 20 and 30 min after a 1-min mouth rinse with 10 ml of 10% sugar solution. This was done using a pH electrode EUTECH™ (ThermoScientific™, Waltham, Massachusetts, USA) connected to an Orion SA 720 pH/ISE Meter (Orion Research Inc., Boston, USA). A total of eight pH registrations, of which four were on coated surfaces and four were on non-coated surfaces, were obtained at each time point. In an attempt to obtain standardized measurements, they were made on the distal portions in cervical areas. The electrode was calibrated against standard pH buffer (Certipur^®^, MERCK, Darmstadt, Germany) at pH 7.00.

### Saliva sampling

The unstimulated (at screening) and stimulated (every visit) salivary secretion rates were measured. Unstimulated saliva was obtained by drooling for 5 min. The stimulated saliva was collected by chewing on a piece of paraffin and continuously spitting out saliva into a beaker for 5 min. Secretion rates were calculated in ml/min. Buffer capacity was assessed using the technique described by Ericsson [18] and determined as final pH.

### Microbial composition for culturing and qPCR

For culturing, pooled plaque samples were collected separately for the different groups of teeth in areas in close contact with the coated sites using a sterile toothpick according to Kristoffersson and Bratthall [19]. Plaque and saliva samples were placed in separate VMGII transport medium until analysed at the laboratory. The samples were dispersed on a Whirlimixer, diluted in tenfold stages in a potassium phosphate buffer and plated in duplicate on Mitis Salivarius agar with bacitracin (MSB) to estimate the mutans streptococci level, Rogosa agar for total lactobacilli count, Mitis Salivarius agar (MS) for total streptococci and blood agar for the total viable count. After incubation in its respective atmosphere for 48–72 h, the number of colony-forming units (CFU) was counted. The number of mutans streptococci was identified by their characteristic colony morphology on MSB agar.

Separate pooled plaque samples for qPCR were collected using a sterile toothpick, placed in an Eppendorf tube containing 100 µl TE buffer and kept at − 80 °C until the analysis. Prior to qPCR analysis, tubes with plaque samples were placed in a thermoshaker (TS-100C, Biosan SIA, Latvia) for 10 min at 95 °C and 1,000 rpm to release genomic DNA. The qPCR relative quantification analysis was performed on a MIC analyser (Bio Molecular Systems, Australia). The reaction mixture of 20 µl in total contained: 1 × qPCRBIO SyGreen mix (PCR BioSystems, London, UK), 400 nM of each forward and reverse primer (Sigma-Aldrich Co., LLC) and 2.5 µl (< 1 µg genomic) DNA template. All the amplifications were carried out in MIC tubes and caps (Bio Molecular Systems, Australia). Detailed information on the primer sequence and qPCR conditions is presented in Table 2.

**Table 2:** qPCR reaction conditions and primer sequences for each evaluated bacteria species

	Primer sequence 5′-3'	qPCR protocol:
*Streptococcus mutans*	Forward: CTACACTTTCGGGTGGCTTG	95 ℃ 2 min,
[24]	Reverse: GAAGCTTTTCACCATTAGAAGCTG	40 × 95 ℃ 10 s, 61 ℃ 20 s
Total *lactobacilli*	Forward: TGGAAACAGRTGCTAATACCG	98 ℃ 2 min,
[25]	Reverse: GTCCATTGTGGAAGATTCCC	40 × 98℃ 10 s, 62 ℃ 15 s
Total bacteria – universal	Forward: TGGAGCATGTGGTTTAATTCGA	94 ℃ 4 min,
[24]	Reverse: TGCGGGACTTAACCCAACA	40 × 94 ℃ 20 s, 62 ℃ 20 s
*Actinomyces naeslundii*	Forward: CTCCTACGGGAGGCAGCAG	94 ℃ 4 min,
[26]	Reverse: CACCCACAAACGAGGCAG	40 × 94 ℃ 20 s, 63 ℃ 20 s
Total *streptococci*	Forward: YGTGCAATTTTTGGATAAT	95 ℃ 3 min,
[27]	Reverse: TTCTATAAGCCATGTTTTGT	40 × Fig. 94 ℃ 20 s, 52 ℃ 30 s

The evaluation of the specificity of each primer was conducted in the reference studies

### Adverse reactions

The soft tissue close to the application sites was evaluated at baseline and on every follow-up visit. In addition, all the participants were asked about any discomfort in connection with the applied material.

### Statistical analysis

The calculations were performed using statistical package software (GraphPad PRISM 7.0A, California, USA). Descriptive statistics (mean value, standard deviation, minimum and maximum values, randomisation) were calculated using Microsoft^®^ Excel, version 15.32. Paired *t* tests and two-way ANOVA with Tukey multiple comparison tests were used to identify differences between and within groups. *p* values of < 0.05 were considered statistically significant.

## Results

A total of 26 subjects completed the study. Two drop-outs were accounted for, one at the 60-day follow-up and the other at the 90-day follow-up, both due to lack of attendance.

### Plaque index

**Fig. 3:**
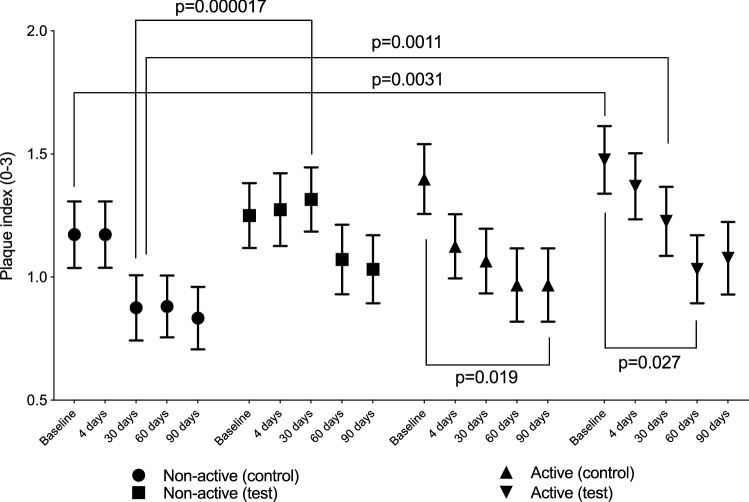
Plaque index (mean, 95%CI) and significant differences observed between and within sites

There was a numerical reduction in the mean plaque index during the 90-day period for the active group, for both the test and control sites (Fig. 3). The reduction followed the same trend at both sites, irrespective of materials applied. A statistically significant reduction in plaque score (*p* < 0.05) was observed for active individuals for test sites between baseline and day 60 and for control sites between baseline and day 90. For the non-active group, a significantly higher plaque score was recorded for the test sites compared with the control sites on day 30.

### Plaque acidogenicity

**Fig. 4:**
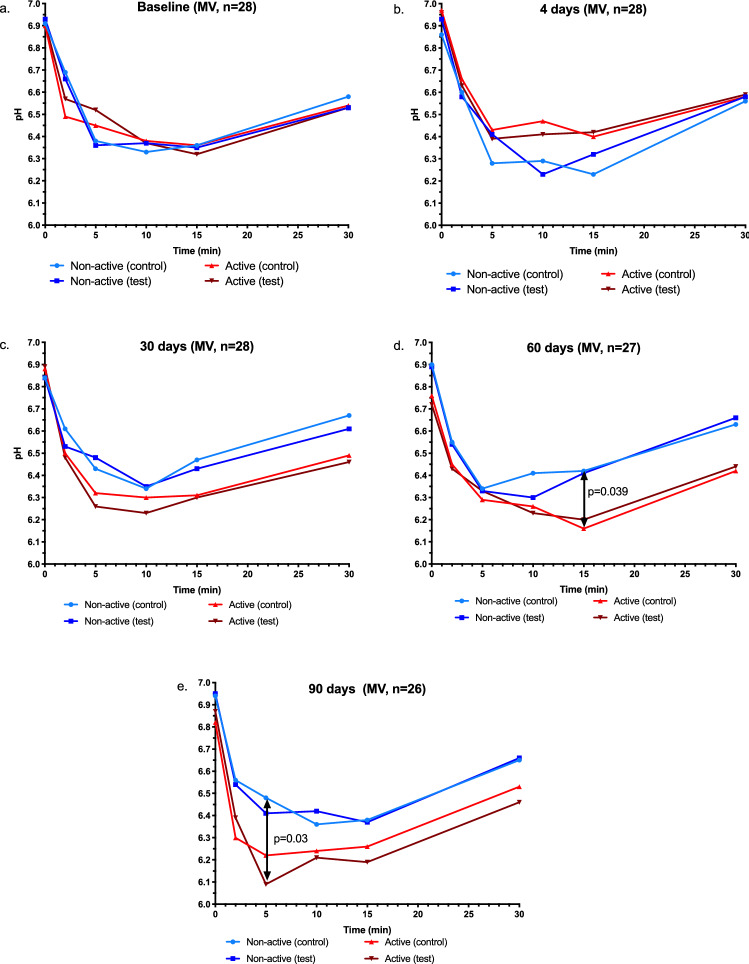
Changes in plaque acidogenicity (mean) after 1-min mouthwash with 10% sucrose solution for separate groups at baseline, 4, 30, 60 and 90 days (**a**–**e**)

Changes in plaque pH for the two sites in the active and non-active group are presented in Fig. 4a–e. At baseline, the two groups and sites resulted in a similar pH pattern, while numerical differences were found on day 4, with a less pronounced pH fall for the active individuals. The opposite pattern with a less pronounced pH fall for the non-active group was found on days 30, 60 and 90. Significant differences were found between the control sites for active and non-active individuals at 15 min on day 60 (*p* < 0.05) and between the control sites in non-active and test sites in active individuals at 5 min on day 90 (*p* < 0.05).

### Buffer capacity

The mean of registered saliva buffer capacity values in both groups was stable over the course of the study. A paired *t* test revealed no significant difference between the non-active and active groups at the respective time points.

### Microbiological composition of saliva and plaque

**Fig. 5:**
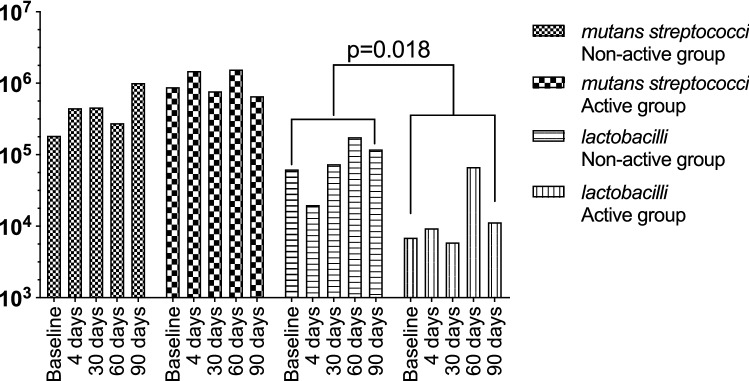
Mean values for *mutans streptococci* and total *lactobacilli* in saliva samples

Salivary levels of mutans streptococci and lactobacilli after culturing are presented in Fig. 5. A variation in bacterial counts was seen between the groups and the different time points. A numerical increase was seen in the non-active group, while no such trend was found for the active group. Significant differences (*p* = 0.018) in the total lactobacilli count were observed between the active and non-active groups.

**Fig. 6:**
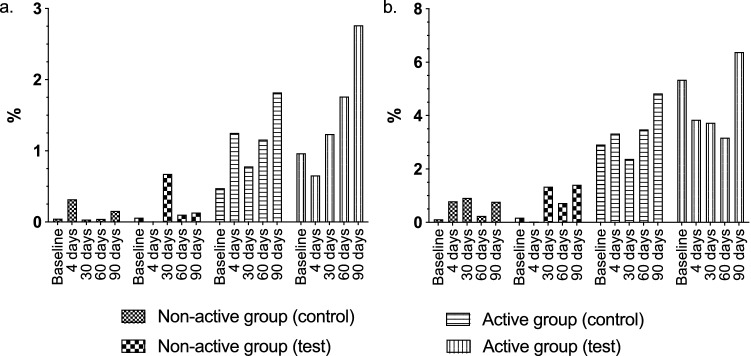
*Streptococcus mutans* prevalence in plaque (**a**) in relation to total bacteria count and (**b**) in relation to total streptococci count

No significant changes were observed in the plaque levels of mutans streptococci in relation to the total number of bacteria (Fig. 6a). In spite of this, the level of mutans streptococci in relation to the total streptococci count displayed a tendency to decline in the active group at the test sites (Fig. 6b). The levels of total streptococci, as well as total lactobacilli, in relation to the total bacterial count remained unchanged throughout the study.

Gene expression analysed by qPCR, used to enumerate the investigated bacterial species, remained stable throughout the study with one exception – *Actinomyces naeslundii*, where the level dropped significantly in the control group on day 4 in relation to the baseline value. The *A. naeslundii* level was raised at 30 days in the non-active group at test sites (*p* = 0.09) and at 60 days in the active group at test sites (*p* = 0.09). Some tendency in the gene expression level in relation to baseline (non-significant 0.1 > *p* > 0.05) was noted, where the *S.mutans* level dropped on day 4 in the active group at test sites (*p* = 0.1), whereas the total streptococci levels on day 4 dropped in the active group at control sites (*p* = 0.058) and increased in the non-active group at test sites (*p* = 0.09).

### Adverse reactions

None of the patients reported any discomfort reaction. No adverse reaction in the soft tissue was observed during the entire test period.

## Discussion

The results from the present study showed that the application of S-PRG significantly reduced plaque accumulation over time. The decrease was evident, both for test vs control subjects and between the test vs control sites and the null hypothesis relating to plaque accumulation was rejected. On the other hand, the null hypothesis regarding pH stabilisation and bacterial composition was accepted, as no significant differences between test and controls (i.e., individuals and sites) could be recorded. A positive trend was seen in the decrease in *mutans streptococci* within the test group at the coated sites, although it was not significant.

Even though *in-vitro* evaluations of the S-PRG barrier coat have shown promising results [8–11, 16], to our knowledge, the present study is the first attempt to evaluate the parameters recorded for the S-PRG barrier coat in vivo. The design of the present study was considered beneficial, as it was conducted as a double-blind, split-mouth, randomised, controlled clinical trial. Using that design, neither the examiners nor the participants were aware of the type of material (i.e., active, non-active) which was applied/received. The importance of this protocol was that it made bias less likely [20, 21]. The performed pre-baseline calibration of the examiners and clear guidelines regarding the parameters evaluated to achieve similar scores was also made in an attempt to minimise the risk of bias. The evaluation of the plaque index using vision and a probe was discussed and checked. To increase the reliability of pH measurements to compile comparable data, a calibrated pH meter was used with calibration performed before each measurement. Because of a slight fluctuation in the values during measurements, due to the small measurement surface area, it was decided to use the value at each measurement that was stable for three seconds.

Difficulties relating to the handling of the material were experienced during the trial. This applied in particular to the mixing procedure before application. To avoid compromising the potential effect of the material, a correct blend is needed. In that respect, the container in which the material was delivered cannot be considered ideal for that purpose. The risk of incorrect mixing was evident, as the amount of base did not appear to be constant on every occasion. It can be speculated that the results that were obtained could have been more pronounced if a more satisfactory mixing process had been performed. In this case, the applied material could have been more homogeneous and its adhesion to the tooth surface might have increased. In the case of the latter, the examiners found that the material partly deteriorated with time. It is, therefore, possible to speculate about whether the etching of the tooth surface using a mild etching agent before the application of the S-PRG barrier coat could have had additional positive effects as a result of a longer retention time for the material.

A change in the recovery of pH to baseline was found when the active group vs the non-active group were compared. A faster recovery of pH was observed for the active group on day 4. On day 30, a slower pH-stabilisation time was shown compared with the non-active group and, on days 60 and 90, the control sites (non-coated) in the active group expressed a lower pH in comparison to the non-active group. The reason has to be further investigated, but, from the present study, it is possible to suggest that the individual differences in pH between the active and non-active groups were less from a caries-prevention perspective. This is also indicated by a decrease in pH above 6 [5, 22].

While analysing the bacterial composition, some interesting variations were recorded. The percentage of *mutans streptococci* increased over time in comparison to the total number of bacteria, while they decreased in percentage terms in comparison to the total number of *streptococci.* This indicated a shift in the plaque composition of *streptococci.* Moreover, subjects in the active group displayed a decrease in the total number of bacteria, especially at the test sites (coated). It is possible to speculate about whether the release of boron (B) ions from the S-PRG particles could explain the results that were obtained. Miki et al. [9] found that boron has a significant impact on reducing the number of *mutans streptococci*. In addition, Kitagawa et al. [10] found that boron, as well as fluoride, had a significant effect on the metabolism of *mutans streptococci*. The leakage of neither B^−^ nor F^−^ was evaluated in the present study. Nonetheless, supported by the findings from other studies, the release of B^−^ and F^−^ from S-PRG particles in the coating material could explain the trend towards a shift in *streptococci* at the test (coated) site found in the present study [9, 10, 15]. Yoshihara et al. [23] found that, despite the release of ions from S-PRG particles, no effect on *mutans streptococci* could be seen and those micro-organisms were located at sites of the particles with etch pits created by lactic acid. They explained the findings as the insufficient release of ions from the particles, as well as a change in surface structure that promoted biofilm formation [23].

The present *in-vivo* study of an S-PRG coating material revealed somewhat differing results in terms of plaque accumulation. The accumulation decreased in the active group compared with the non-active group, with a significantly lower plaque index between baseline and day 90. A lower mean plaque index was recorded at the test (coated) sites in the active group at each follow-up visit, a finding that was not seen in the non-active group. Even the untreated control sites in the active group showed a similar, albeit not significant, decrease in plaque index. This finding could be an indication that the active substances in the test material had an effect on plaque accumulation, since the non-active group did not show this decrease. Moreover, in the non-active group, the test (coated) sites displayed an increased plaque index until day 30. Since the only difference between the active and non-active material was the inclusion of the active S-PRG particles in the active material, this could indicate that the non-active material placed in the non-active group acted as a retention site for bacteria. In contrast, on the test (coated) site in the active individuals, the active material created an environment for a stable reduction in the amount of plaque recorded. These results are in accordance with previous *in-vitro* results [9, 10]. It was, therefore, suggested that the active ingredients in the S-PRG barrier coat reduced plaque accumulation; otherwise, both the control- and test-coated sites would have followed a similar trend. Furthermore, it can be assumed that most of the material remained on the teeth up until 30 days because of the significant difference between the groups. Based on the results obtained, it can also be speculated that the active substances had an effect on plaque accumulation up to 60–90 days because of the trend towards reduced accumulation in the active group. This reduction could not be solely ascribed to toothbrushing or improved oral hygiene, as, in that case, both groups would have followed similar trends. So, validated by the results in the present study, the null hypothesis regarding “no difference in plaque accumulation” could be rejected.

The incorporation of active ion-releasing particles in coating materials appears to have a positive effect on plaque accumulation up to 60–90 days. In spite of this, complementary clinical studies are needed to further evaluate the caries-prevention potential of materials containing S-PRG particles. In the present study the majority of volunteers were females. There was no special reason for this as the subjects were strictly consecutively included into the study. In addition, no differences in plaque pH and buffer capacity affected by age and gender were found. Future studies should preferably be performed on larger populations with a more varied caries situation and broader age and gender groups. Since the material includes methacrylates, its potential for adverse side effects was additionally evaluated in the present study (data not shown). However, no adverse effects during the observation time for either of the examined groups were recorded.

Within the limitations of the present study, the following conclusions can be drawn.

1. The test material showed a significant decrease in plaque accumulation over time (60 days) compared with the non-active material. The null hypothesis was, therefore, rejected.
2. The studied material showed no significant change in either pH stabilisation or bacterial composition. The null hypothesis was, therefore, accepted.
